# Role of anesthesiology curriculum in improving bag-mask ventilation and intubation success rates of emergency medicine residents: a prospective descriptive study

**DOI:** 10.1186/1471-227X-11-8

**Published:** 2011-06-16

**Authors:** Hassan Soleimanpour, Changiz Gholipouri, Jafar Rahimi Panahi, Mohammad Reza Afhami, Rouzbeh Rajaei Ghafouri, Samad EJ Golzari, Maryam Soleimanpour, Robab Mehdizadeh Esfanjani

**Affiliations:** 1 Emergency Medicine Department, Tabriz University of Medical Sciences, Daneshgah Street, Tabriz 51664, Iran; 2 Anesthesiology Department, Tabriz University of Medical Sciences, Daneshgah Street, Tabriz 51664, Iran; 3 Gastroenterology Research Centre of Tabriz University of Medical Sciences, Tabriz, Iran; 4 Department of Statistics, Tehran North Branch, Islamic Azad University, Tehran, Iran

**Keywords:** Education, Curriculum, Anesthesiology, Emergency Medicine

## Abstract

**Background:**

Rapid and safe airway management has always been of paramount importance in successful management of critically ill and injured patients in the emergency department. The purpose of our study was to determine success rates of bag-mask ventilation and tracheal intubation performed by emergency medicine residents before and after completing their anesthesiology curriculum.

**Methods:**

A prospective descriptive study was conducted at Nikoukari Hospital, a teaching hospital located in Tabriz, Iran. In a skills lab, a total number of 18 emergency medicine residents (post graduate year 1) were given traditional intubation and bag-mask ventilation instructions in a 36 hour course combined with mannequin practice. Later the residents were given the opportunity of receiving training on airway management in an operating room for a period of one month which was considered as an additional training program added to their Anesthesiology Curriculum. Residents were asked to ventilate and intubate 18 patients (Mallampati class I and ASA class I and II) in the operating room; both before and after completing this additional training program. Intubation achieved at first attempt within 20 seconds was considered successful. Successful bag-mask ventilation was defined as increase in ETCo_2 _to 20 mm Hg and back to baseline with a 3 L/min fresh gas-flow and the adjustable pressure limiting valve at 20 cm H_2_O. An attending anesthesiologist who was always present in the operating room during the induction of anesthesia confirmed the endotracheal intubation by direct laryngoscopy and capnography. Success rates were recorded and compared using McNemar, marginal homogeneity and paired t-Test tests in SPSS 15 software.

**Results:**

Before the additional training program in the operating room, the participants had intubation and bag-mask ventilation success rates of 27.7% (CI 0.07-0.49) and 16.6% (CI 0-0.34) respectively. After the additional training program in the operating room the success rates increased to 83.3% (CI 0.66-1) and 88.8% (CI 0.73-1), respectively. The differences in success rates were statistically significant (P = 0.002 and P = 0.0004, respectively).

**Conclusions:**

The success rate of emergency medicine residents in airway management improved significantly after completing anesthesiology rotation. Anesthesiology rotations should be considered as an essential component of emergency medicine training programs. A collateral curriculum of this nature should also focus on the acquisition of skills in airway management.

## Background

Effective airway management is the main part of emergency resuscitation, with many seeing it as an indisputable core skill for emergency physicians (EPs) [1]. However, an earlier study on ED intubations reported an alarmingly high complication rate for orotracheal intubations [2].

The current basic skills, listed in trainees' logbooks seem to be insufficient. This indicates that a closer co-operation between emergency medicine and anesthesiology is required, starting from collegiate level and extending to departmental levels [3].

It is important not to try to turn EPs into anesthetists, but instead equip them with the skills they need for their particular environment and the problems they face [4].

Since concurrent failure of orotracheal intubation and mask ventilation can ultimately result in death or brain damage, these two basic techniques are the most important skills an anesthetist learns [5].

Although there are seemingly numerous opportunities for EM residents to learn these skills outside the operating room, they must also be given the opportunity to practice these maneuvers in a controlled setting of an operating room.

Anesthesiology rotation is an important component of EM training that should focus on the acquisition of airway skills [6].

For the past 25 years, higher specialist trainees in A and E Medicine in the UK have been required to complete a minimum of 3-month secondment in anesthesia and intensive care [7]. Although during their training period in the skills lab and operating room trainees have an opportunity to develop specific learning objectives for airway management, our research provides them with the opportunity to focus on two of the most important skills: Bag mask ventilation and orotracheal intubation.

The aim of this study was to determine the efficacy of the anesthesiology curriculum on the success rates of bag-mask ventilation and orotracheal intubation performed by EMRs.

## Methods

A prospective descriptive study was conducted at Nikoukari Hospital - a teaching hospital located in Tabriz, Iran.

Our EMRs were trained in a skills lab on dummies and were then asked to bag-mask ventilate, and intubate patients in the operating room before and after an additional anesthesiology training program. They were asked to perform the procedures as part of their normal training program.

The anesthesiology curriculum had already been approved by our university for emergency residents as part of their residency program, which they were obliged to attend.

It should be mentioned that there is a mandatory one-month rotation in anesthesia during the first year of the EM residency (EMR-1). During this one month period, the EMR-1s learn airway management and perform orotracheal intubations on stable patients in the operating room.

Although our study was designed to describe the efficacy of the anesthesiology curriculum and it was not an interventional study; ethical approval was obtained. Furthermore, all the ED residents who took part in the curriculum were already qualified in bag-mask ventilation and orotracheal intubation, since they had already passed the required training courses in the Skills Laboratory and attained their certificates.

A total number of 18 EMR-1s received traditional instruction about orotracheal intubation and bag-mask ventilation in the skills lab, using mannequin-based simulators for a period of 36 hours. The training program in the skills lab included a course of theoretical instruction that EMR-1s received regarding orotracheal intubation and bag-mask ventilation. Power Point software and video-based methods were employed in the program. As hands-on training, the residents were then asked to bag-mask, ventilate, and intubate the mannequins for at least 20 times. The steps required in performing these procedures successfully were instructed by an attending anesthesiologist, who also dealt with the theoretical aspects. The theoretical and hands-on training portions in this 36-hour course were approximately equal. All of the participants passed a qualification exam.

As part of an anesthesiology rotation, the same group was trained in airway management in an operating room over a one-month period. During this period, EMR-1s received an extensive didactic review of airway management, simple airway maneuvers as well as bag-mask ventilation and orotracheal intubation. The rotation also included the basic skills of airway assessment, mask ventilation, orotracheal intubation and airway decision-making. In order to pass the curriculum successfully and as their hands-on training, the residents needed to bag-mask, ventilate and intubate at least 50 patients in the operating room.

In our research, the residents were asked to bag-mask, ventilate, and intubate 36 adult patients (18-52 year-olds) in the operating room both before and after the one-month anesthesiology rotation. Each resident performed both procedures on 2 patients.

The selected patients had Mallampati class I and ASA class I and II. The exclusion criteria were: 1 - presence of beard, 2 - edentulousness, 3 - facial anomalies, 4 - having a nasogastric tube, 5 - morbid obesity and a history of snoring. Patients undergoing elective ophthalmic surgery were aware of attending a teaching hospital and they willingly participated in this medical study.

Written informed consents were obtained from the patients before admission with an understanding that there would be students working on their cases as part of an ongoing experiment since Nikookari Hospital is a teaching hospital.

For all intubations, patients were connected to cardiac monitors, automated blood pressure monitors, pulse-oximeters and capnography monitors. An attending anesthesiologist supervised the procedures at all times.

All patients were hydrated preoperatively with Ringer's Lactate solution 10 mL.kg^-1^. After pre-oxygenation for 3 minutes and premedication with midazolam 0.02 mg.kg^-1 ^and fentanyl 1.0 μg.kg^-1^, anesthesia was induced with propofol (2 mg.kg^-1^) and atracurium (0.5 mg/kg).

When the patients became unconscious, as judged by loss of response to command and loss of eyelash reflex, mask ventilation was initiated.

The total fresh gas flow (FGF) on the anesthetic machine was set at 3 L/min and the adjustable pressure limiting (APL) valve at 20 cm H_2_O. A standard circle circuit and 2 L bags were used. In applying bag-mask ventilation tight mask seal and appropriate compression of the bag was taken into account [8].

The end point for successful bag-mask ventilation was defined as an ETco2 trace increasing to 20 mm Hg and back to baseline. If this was achieved at a total fresh gas flow of 3 L/min and an APL valve at 20 cm H_2_O, bag-mask ventilation was considered to be successful as the primary outcome. If this was not achieved, it was then mandatory to use ancillary techniques to ensure adequate bag-mask ventilation. These techniques were defined as a secondary outcome and included the increasing of FGF to 6 L/min, closure of the APL valve to 30 cm H_2_O, and use of the oxygen flush device and two-person technique (the resident using two hands to secure the mask while an assistant squeezing the bag) [9].

After 3 minutes the trachea was intubated with an appropriate size orotracheal tube.

A successful orotracheal intubation was firstly confirmsed by direct laryngoscopy, secondly by chest rise and auscultation and finally by capnography. The intubation was also considered successful when it was performed on the first attempt and within 20 seconds. Intubation and bag-mask ventilation success rates were recorded by the supervising anesthesiologist. The time period needed for intubation was defined as the time from the cessation of bag-mask ventilation to the time of the confirmation of successful tracheal tube placement which was also recorded by the same supervising anesthesiologist [10].

When the time exceeded 20 seconds, the procedure was aborted and intubation was performed by the supervising anesthesiologist.

The same attending anesthesiologist was always present in the operating room throughout the procedures. He had direct responsibility for all intubations performed in the operating room and had the discretion to determine which resident perform the ventilation and intubation and which method be used.

Success rates in both bag-mask ventilation and orotracheal intubation were recorded and compared both before and after anesthesiology rotation.

The data were analyzed using SPPS version 15. Nominal scale data were reported as absolute and relative frequency and continuous scale data were reported as mean ± SD. To detect differences between before and after education, data were analyzed by McNemar and marginal homogeneity tests for nominal variables. To compare continuous variables, paired t-test we used. P < 0.05 was considered to be statistically significant. The total census of the ED residents was included since the department was newly established and this made the sample size of the study rather small.

## Results

**Figure 1:**
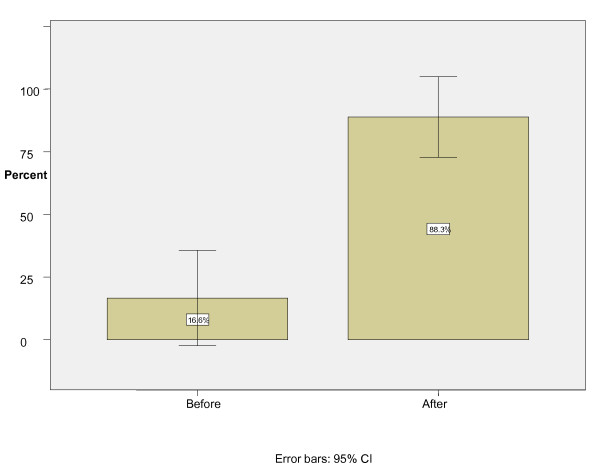
**Bag-mask ventilation success rate before and after instruction**.

**Figure 2:**
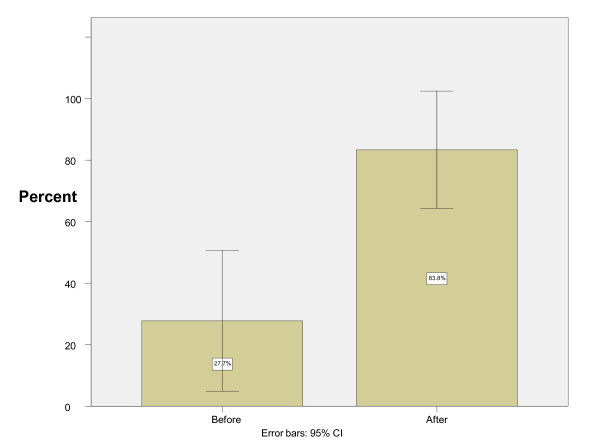
**Intubation success rate before and after instruction**.

There were eighteen EMR-1s who performed both bag-mask ventilation and orotracheal intubation on 36 patients at the beginning and end of the anesthesiology rotation. All the patients were male, with the mean age of 37 years. Before the anesthesiology rotation, the participants had a successful bag-mask ventilation rate of 6 out of 36 (95% confidence interval = 0-34%) and an intubation success rate of 10 out of 36 (95% confidence interval = 7-49%). After the rotation, successful bag-mask ventilation was reported in 32 out of 36 cases (95% confidence interval = 73-100%) and successful intubation in 30 out of 36 (95% confidence interval = 66-100%) cases (Figures 1 &2).

The differences in successful bag-mask ventilation and orotracheal intubation before and after the rotation were statistically significant, P = 0.0004 and P = 0.002 respectively.

In thirty out of 36 patients in which bag-mask ventilation was unsuccessful, ventilation had to be secured using ancillary techniques. The number of failures decreased to only 4 after the completion of anesthesiology curriculum by ER residents (Tables 1 &2). The use of ancillary techniques to provide adequate bag-mask ventilation was reduced after the anesthesiology rotation and there was a statistically significant difference before and after the rotation (P = 0.001).

**Table 1:** Primary and secondary outcomes in bag-mask ventilation

	Before rotation	After rotation
Primary successful bag-mask ventilation	6 (16.6%)	32(88.8%)

Increasing the FGF to 6 L/min	10 (27.7%)	2(5.5%)

Closure the APL valve to 30 cm H2o	10 (27.7%)	2(5.5%)

Using the oxygen flush device	8 (22.2%)	0

Using a two-person technique	2 (5.5%)	0

Total	36	36

**Table 2:** Frequency of failed endotracheal intubation

Failed endotracheal intubation	Before rotation	After rotation
Prolonged attempt	20	4

Esophageal intubation	6	2

Total	26	6

The average time spent on successful orotracheal intubation was 18.6 ± 1.67 seconds before anesthesiology rotation, but this value decreased to 13.6 ± 1.34 seconds at the end of the rotation in the same group (P = 0.043).

## Discussion

With the development of emergency medicine as a recognized medical specialty, emergency airway management has become an essential skill for emergency physicians. There has been remarkably little literature describing the airway management skills for emergency physicians. We undertook this study to determine the impact of a one-month anesthesiology rotation on improving airway management skills of EMR-1s. The only set of specific objectives of an anesthesiology rotation to be achieved by an emergency medicine trainee has been published in the United States of America [11].

Amarasinghe et al.^6 ^have identified the core components of an Anesthesiology curriculum for emergency medicine trainees, and demonstrated that the most important skills to be learned on an anesthesiology rotation are orotracheal intubation, bag-mask ventilation, jaw thrust/chin lift maneuver, and the use of oral and nasal airways.

Based on the results of Amarasinghe's study, our research focused on assessment of the two most important and highly useful airway management skills; bag-mask ventilation and orotracheal intubation.

We observed that most residents who received traditional instructions regarding airway management in the skills lab using mannequin-based simulators could not manage the patient airway successfully. They had difficulty ventilating and intubating patients with relatively easy airways in the operating room setting, even though all of participants had passed a certification exam. Considering the significant acquisition of airway management skills after human-based instruction, we believe it is necessary to use this method along with traditional mannequin-based training.

It has recently been suggested that future training of UK EM-specialists should include a one-year anesthesia and critical-care rotation, involving 6 months of full time anesthesia followed by 6 months of full time critical care rotations [12]. Another study showed that anesthesiology rotation is an essential part of EM training and that the optimal duration is approximately 6 months, preferably in the first 2 years of advanced training [6].

The duration of the rotation on acquisition of airway management skills is probably not long enough and should be extended. In the future it is also important to study the effects of increased length and quality of training programs for EPs on the success and failure rates for airway management carefully in the ED.

It should also be remembered that the acquisition of anesthesiology-related knowledge and skills is not confined to an anesthesiology rotation. In fact, many of these may be learned more effectively in other settings. For example, knowledge of the pharmacology related to this area is an important part of the primary examination curriculum. Intubation of trauma and unstable patients may be best learned in EDs under appropriate supervision, because these patients differ from those encountered in elective anesthetic practice [6].

It is widely accepted that orotracheal intubation in the emergency room is significantly more hazardous and is more frequently associated with an increased rate of difficult intubation and failed intubation than in the operating room [13].

Maintenance of skills in emergency airway management is also currently a subject of considerable debate. There is no objective scientific data to support a minimum requirement of numbers of emergency or rapid sequence intubations (RSI) to be performed by EPs (or indeed anesthetists) to maintain competency in this area.

The figures from the Trauma Audit Research Network suggest a maximum of approximately 6 RSIs per month and per department (based on an average of 68,000 patients per year) which if staffed by 4 consultants in EM equates to 1-2 RSIs per consultant per month. The Scottish Trauma Audit Group data also suggest that the individual consultant in EM is likely to be involved in 2-3 RSIs per month [7]. Due to the presence of a large number of patients at our ED (nearly 6000 patients per month); the individual skills maintenance will not be difficult for the practicing EMRs.

In the US, several studies have reported on the requirements and experience of their residents in Emergency Medicine. Although accepting that there are always variations between people and it is difficult to be clear on exact numbers, it would seem that novice anesthesiology residents require 80 or more intubations to achieve reasonably consistent skills in orotracheal intubation [7].

Hayden and Panacek reported that the mean number of intubations per trainee was 75 (95% confidence intervals 62 to 87); this is over a three-year residency in emergency medicine in a US setting [14].

More studies should be carried out to determine the number of orotracheal intubations required to achieve an acceptable level of procedural competency.

## Limitation

This study had some limitations that should be considered when interpreting the results. Our sample size was small and suboptimal. Further prospective studies need to be completed in larger scales to validate the role of such rotations.

Another limitation was the fact that certain complications were not assessed. There was certainly the potential for selection bias that could have been responsible for the differences noted in these rates. Also, the study was completed on patients with relatively easy airways, raising the possibility that the results could not be generalized.

### Other limitations

• The experiment had to be confined to a single site.

• The number of participants was small and limited.

• The duration of Skills Lab program is only 36 hours whereas our studied anesthesiology rotation is one month long. Better results are more likely to be obtained from studies carried out in longer periods.

• The study lacked control group.

• We did not have adequate number of attempts to achieve reasonably consistent skills in bag-mask ventilation or orotracheal intubation.

## Conclusions

Since EMRs' success rate in airway management improved after rotating on an anesthesiology rotation, anesthesiology rotations could be considered as one of the crucial components of EM training programs. We believe that a standardized theoretical instruction program in combination with a practical anesthesiology rotation improve the skills of airway management in EMRs. Airway management training is a continuous process that should begin with theoretical instruction, continue in the skills lab and operating theatre and end in the ED. All of the above mentioned steps should be supervised by an attending anesthesiologist and/or EP

## Abbreviations

A and E: Accident and Emergency; ED: Emergency Department; EP: Emergency Physician; EM: Emergency Medicine; EMR: Emergency Medicine Residents; RSI: Rapid Sequence Intubations; UK: United Kingdom

## Competing interests

The authors declare that they have no competing interests.

## Authors' contributions

HS and CG collected clinical data, reviewed the literature on the topic, and drafted the manuscript. JRP, RRG and MRA conceived of the study, and participated in its design and coordination. SEJG, MS and RME participated in the design of the study and performed the statistical analysis. All of the authors were involved in patient management or the writing of the manuscript. All authors read and approved the final manuscript.

## Pre-publication history

The pre-publication history for this paper can be accessed here:

http://www.biomedcentral.com/1471-227X/11/8/prepub
